# A 3D-printed adult release system compatible with a drone for aerial deployment of *Aedes aegypti* and *Glossina palpalis gambiensis*

**DOI:** 10.1186/s13071-025-07146-7

**Published:** 2025-11-27

**Authors:** Hamidou Maiga, Anibal Morales Zambrana, Nanwintoum Sévérin Bimbilé Somda, Wadaka Mamai, Thomas Wallner, Simran Singh Kotla, Hanano Yamada, Ricardo Antonio de Oliveira Machado, Nicholas Rodwell Matias, Abdoulaye Diabaté, Jérémy Bouyer, Chantel Janet de Beer

**Affiliations:** 1 Insect Pest Control Section, Joint FAO/IAEA Centre of Nuclear Techniques in Food and Agriculture, Department of Nuclear Sciences and Applications, International Atomic Energy Agency, Vienna, Austria; 2 Institut de Recherche en Sciences de La Santé, Direction Régionale de L’Ouest (IRSS-DRO), Bobo-Dioulasso, Burkina Faso; 3 Unité de Formation et de Recherche en Science et Technologie (UFR/ST), Université Norbert ZONGO (UNZ), BP 376, Koudougou, Burkina Faso; 4 Birdview DroneBioControl, São Paulo, Brazil; 5 ASTRE, CIRAD, INRAE, Université de Montpellier, Plateforme Technologique CYROI, Sainte Clotilde, La Réunion France

**Keywords:** Unmanned aerial vehicle, Drone, Insect releases, Sterile insect technique, Mosquito, Tsetse

## Abstract

**Background:**

The sterile insect technique (SIT) is a well-established, environmentally friendly method of insect population suppression that relies on the release of sterile males to reduce reproduction in wild populations. SIT has been successfully applied against several insect pests, including the tsetse fly (hereafter tsetse) in Africa and *Aedes aegypti* mosquitoes in Asia and the Americas, and is increasingly considered to be a complementary tool for vector control. For an SIT programme to succeed, the release process must ensure good coverage of the targeted area without compromising the performance of the released insects. The use of release systems paired with drones may play an important role. While interest in aerial releases is growing, the number of available aerial release systems remains limited.

**Methods:**

The Birdview insect cassette, a lightweight, three-dimensional (3D)-printed device compatible with drones, was described and assessed for its suitability to release adult *Aedes* mosquitoes and tsetse under laboratory conditions. We determined the carrying capacity of the release system and the flight propensity, survival of and potential physical damage to *Ae. aegypti* and the tsetse *Glossina palpalis gambiense*, under laboratory conditions, using between 8000 and 30,000 insects.

**Results:**

Overall, our findings highlight the potential of the insect cassette system, which can support loading densities up to 45,000 *Ae. aegypti* or 11,000 *G. p. gambiensis*, with release efficiencies ranging from 60% to 96% and a survival rate of > 70% after a 26-day monitoring period. Of the released insects, > 80% escaped from flight ability devices.

**Conclusions:**

The Birdview insect cassette is valuable for aerial release programmes targeting *Aedes* mosquitoes and tsetse. Future research should focus on refining the system's design and functionality, as well as evaluating its performance in field settings to validate its effectiveness in vector control.

**Graphical Abstract:**

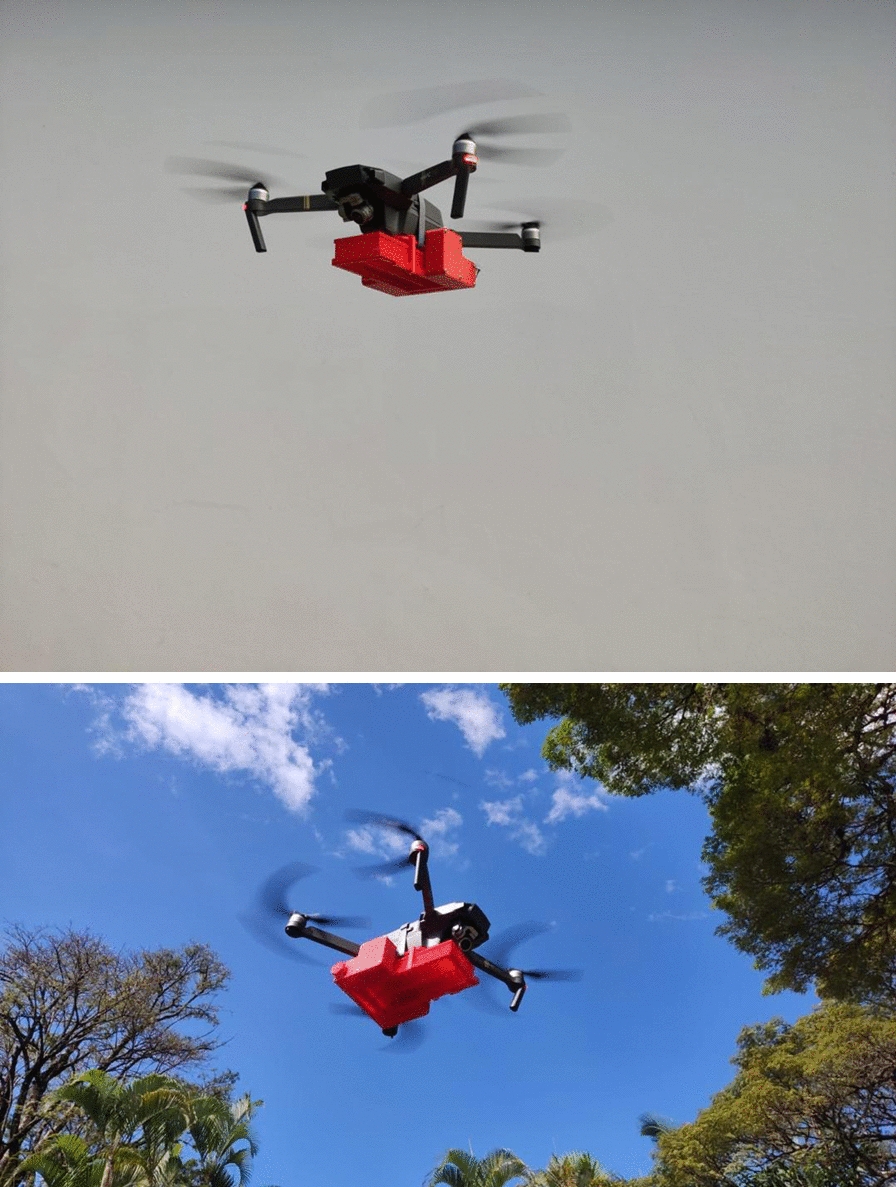

**Supplementary Information:**

The online version contains supplementary material available at 10.1186/s13071-025-07146-7.

## Background

Vector-borne diseases such as dengue, yellow fever, chikungunya and African trypanosomosis are transmitted by mosquitoes and tsetse flies (hereafter tsetse). Both mosquitoes and tsetse have played a major role throughout human history from the Paleolithic era to the modern age through a variety of mechanisms that include but are not limited to killing humans with demographic and population level impacts and changing human relationships with the environment [1]. Vector-borne diseases currently account for > 17% of all infectious diseases and seven million deaths annually in humans [2]. Dengue is currently the most prevalent viral infection transmitted by *Aedes* mosquitoes, and > 3.9 billion people across more than 129 countries are at risk [2]. African animal trypanosomosis, transmitted by tsetse, is also a major obstacle to rural development and to the adoption of more efficient and sustainable agricultural production systems in Sub-Saharan Africa [3]. Chemical-based methods are regularly used to manage *Aedes* mosquitoes and tsetse populations (*Glossina* spp.) [4, 5]. However, these control measures come with a cost as they can reduce biodiversity, destroy habitats [6], and for mosquitoes, promote and develop insecticide resistance in the targeted populations [7]. Therefore, a more selective use of chemical-based methods within an integrated pest management strategy has been emphasized and adopted.

The sterile insect technique (SIT), a component of the area-wide integrated pest management (AW-IPM) approach, has been recognized as a promising method that is suitable for use in integrated pest management programmes to manage insect pests and vectors such as fruit flies [8], *Aedes* mosquitoes [9], the New World screwworm [10] and tsetse [11]. The SIT is an environmentally suitable, species-specific, genetic control tactic that requires the rearing of large numbers of the target insect, sterilization of the males with ionizing radiation and the release of large numbers of these sterile males into the vector population on an area-wide basis. The released sterile males then compete with wild males for mating with the wild female population, thereby interrupting their reproductive potential and reducing population numbers over time [12]. For any pest management programme that includes the SIT to be successful, it is crucial that the released sterile males are of good quality [13], have adequate mobility and are not damaged during the release process.

Aerial release systems have been developed and are being used to release tsetse within the framework of operational SIT programmes. Fixed-wing aircraft and gyrocopters that utilize biodegradable cardboard boxes [11] and, more recently, geographic information systems (GIS)-programmable Smart Release Machines that incorporate chilling technology [14, 15] have been used in the past. However, despite the major successes that have been achieved with these aerial release systems for tsetse, they continue to be expensive. For mosquito SIT programmes, there is an increased interest in developing aerial release systems that will reduce work-load and operational costs when release sites are inaccessible, and allow a better dispersal of the released insects [16]. In male release-based strategies, including SIT pilot programmes involving drones, the use of GIS and global positioning systems (GPS) have shown promising applications [17–19], although current limitations include not only payload size but also evolving airspace regulations [20]. The major advantages of using drones include reduced operating costs and improved accuracy in insect dispersal [16], without compromising insect quality. Drones can also provide access to restricted areas where the use of conventional aircraft is not permitted by legislation due to the risk of accidents and environmental concerns [16].

In this article, we have described and assessed the Birdview insect cassette (Birdview DroneBioControl, São Paulo, Brazil), a 3D-printed adult release system compatible with a drone, under laboratory conditions for its suitability for releasing *Aedes aegypti* and *Glossina palpalis gambiensis*. The carrying capacity of the release system and the flight propensity, survival of and potential physical damage to the released *Ae. aegypti* and *G. p. gambiensis* were determined.

## Methods

### The Birdview insect cassette and its release mechanism

**Fig. 1:**
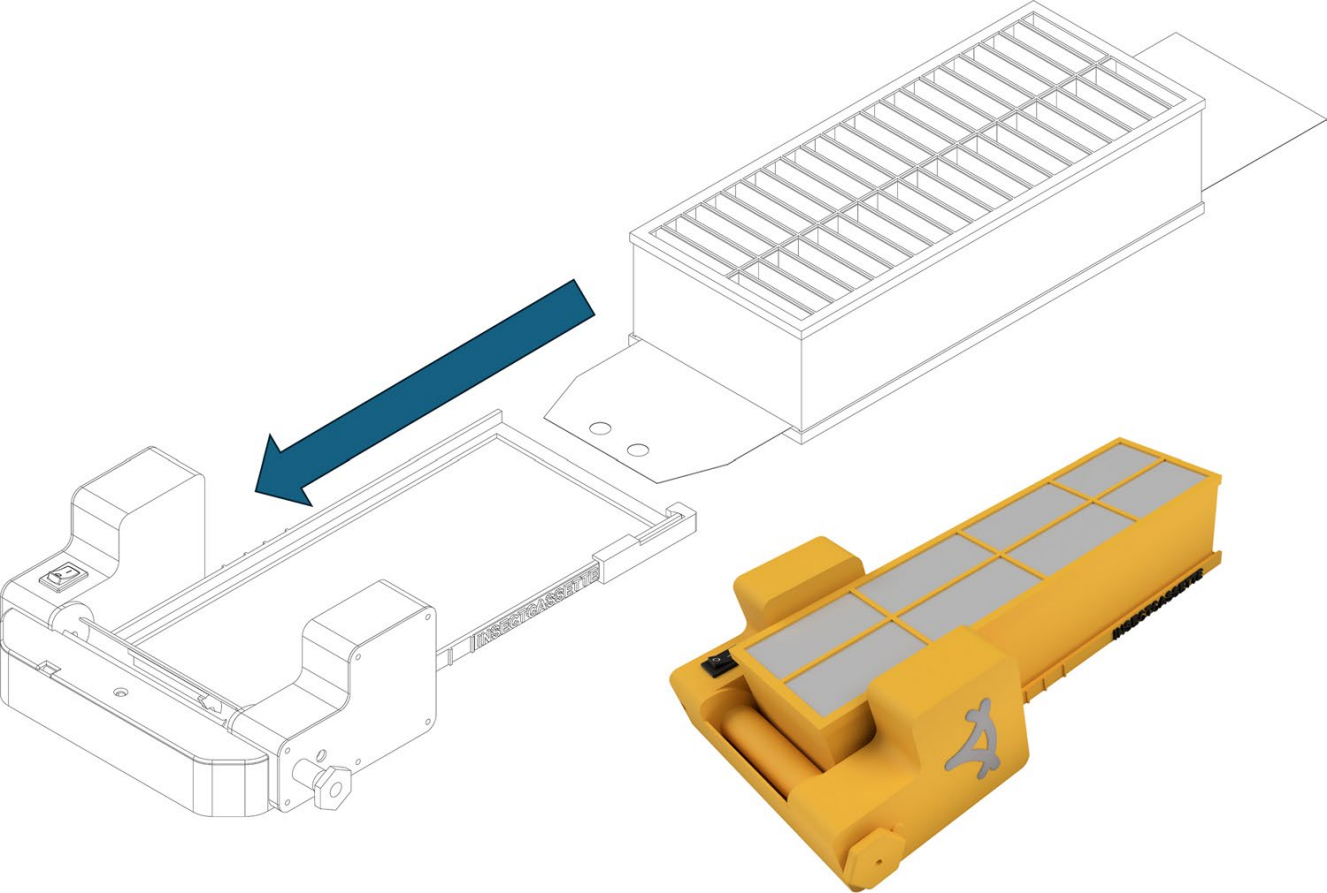
Drawing of the holding frame of the slide cassette assembly into the implement structure, ensuring film passes under the spool and curves upwards, then attaches to the reel (left) and a photograph of the holding frame and a cassette of the Birdview insect release system (right)

**Fig. 2:**
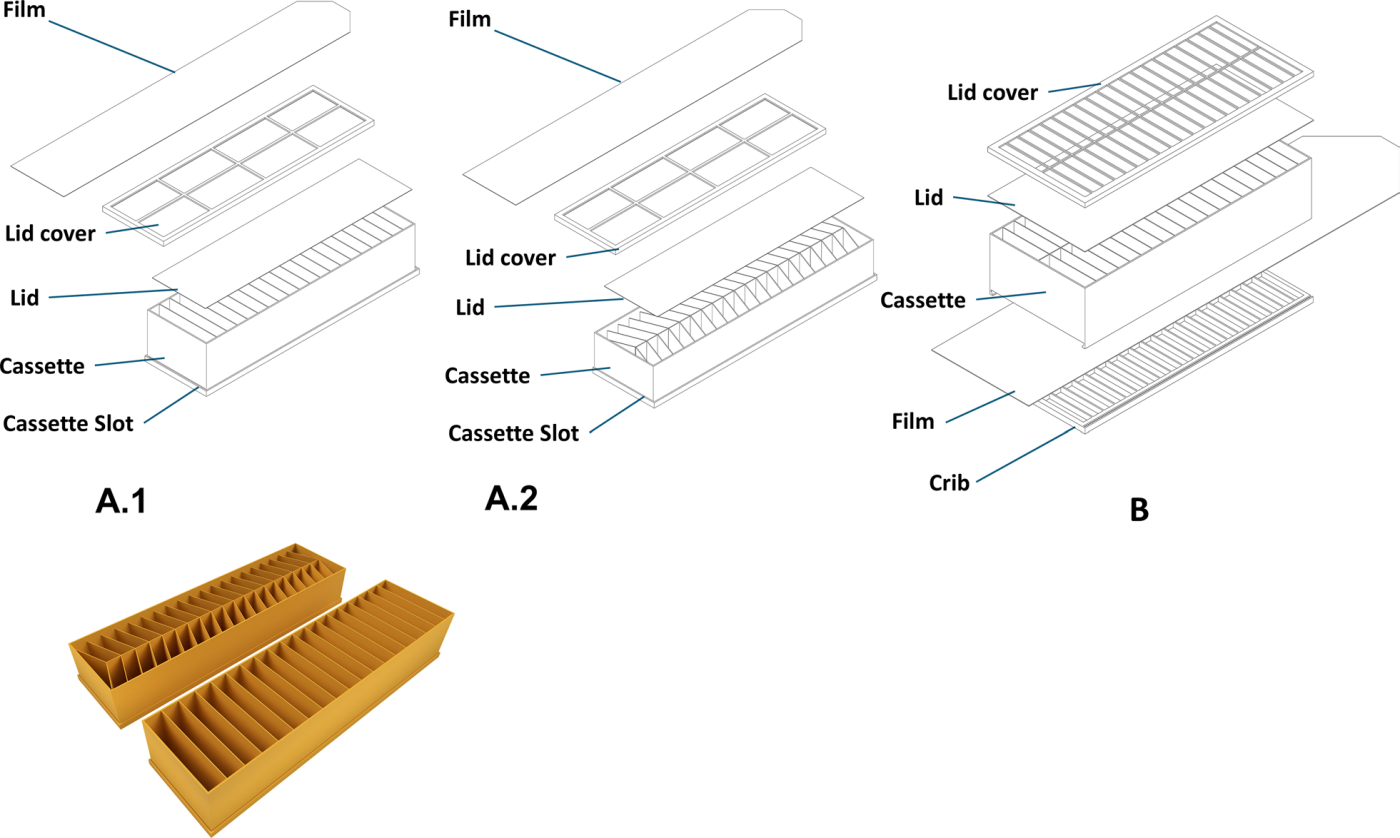
Drawings and photo of the insect cassettes versions A.1 and A.2 and drawings of the cassette that can fit in the insect cassette system version B (manufactured by Birdview DroneBioControl

The Birdview insect release system used in this study (Birdview DroneBioControl, São Paulo, Brazil), consists of a three dimensional (3D)-printed holding frame (Fig. 1) and insect cassettes (Fig. 2). The holding frame houses electronic components, a reel with a thin plastic sheet and a cassette housing. The insect cassette, also 3D-printed, holds insects for release. Both parts are made from polylactic acid (PLA), a biodegradable thermoplastic from renewable resources, and use polypropylene sheets to cover cassette openings. We assessed different versions of the insect cassette system:

Version A (A.1, A.2). The holding frame weighs 149 g and supports two cassette types. The cassette type in version A.1 has dimensions: 21.1 × 4.4 × 6.0 cm; 76 g; 20 cells (1.0 × 4.0 × 5.8 cm each); internal volume 464 cm^3^; system weight 225 g. The cassette type in version A.2 has dimensions: 21.1 × 4.4 × 5.8 cm; 60 g; 38 cells (3.1 × 3.1 × 1.0 cm); internal volume 365 cm^3^; system weight 209 g. In both version A.1 and A.2, cells are covered by a transparent sheet and lid on top, with a bottom sheet attached to the reel. When powered, the reel retracts the bottom sheet, sequentially uncovering cells for insect release. Version A runs on a lithium-polymer battery (2S 7.4 V, 300 mAh) connected to an Arduino Pro Mini and a DS04-NFC servomotor, with a runtime of 692 s (11 min 32 s).
Version B. The holding frame weighs 193 g, and the cassette weighs 119 g (312 g total). The cassette (21.4 × 5.0 × 9.0 cm) has 40 cells in two rows (1.0 × 4.5 × 4.3 cm each), with a total internal volume of 774 cm^3^. A black top sheet blocks light, guiding insects downward, while a transparent bottom sheet attached to the reel enables sequential release. The same battery as used in versions A.1 and A.2 also powers version B, but the latter uses a D1 Mini ESP8266 microcontroller with Wi-Fi, connected to the DS04-NFC servomotor. Release speeds are configured via smartphone, with three preset modes are accessible through a slide switch. Runtime varies from 180 s (fastest) to 1,114 s (slowest, 18 min 34 s).

Both of these insect cassette system versions can be integrated into drones for aerial releases.

### Biological material and experiments

#### Insect cassette suitability and release process impact assessment of *Ae. aegypti*

##### Strain rearing and handling

The *Ae. aegypti* strain used in the study was transferred from the insectary of Biofabrica Moscamed (Juazeiro, Brazil) in 2012 to the Insect Pest Control Laboratory (IPCL) of the joint FAO/IAEA Center of Nuclear Sciences and Applications (Vienna, Austria). It was maintained following modified mass-rearing procedures as described in [21, 22], which built upon and refined the standard mass-rearing procedures developed at the IPCL [23]. The larval and adult stages were reared at temperatures of 28 ± 1 °C and 26 ± 2 °C and relative humidity (RH) of 80% ± 10% and 60% ± 10%, respectively. Both larvae and adults were kept under a 14:10-h (light:dark) photoperiod that included 1 h of dawn and 1 h of dusk.

To allow adult emergence for this experiment, 2000 male pupae were sorted using an automatic pupae sex separation system [23, 24] and aliquoted into 600-ml plastic cups, which were placed in insect cages (30 × 30 × 30 cm) (BugDorm, MegaView Science Co., Ltd., Taichung, Taiwan) for emergence. Adult mosquitoes were maintained with access to a 10% sucrose solution until the day of the experiments.

To assess the Birdview insect cassette (versions A.1/A.2), we placed adult male *Ae. aegypti* in a cold room at 4–5 °C for 10 min to achieve knockdown for the chilling and compaction procedures following a previously described protocol [25]. In short, after the knockdown, males were removed from the cage by gently tapping the cages, resulting in the males dropping onto a paper towel that lined a 40 × 30 × 8-cm tray. The males were then transferred into the Birdview cassette (version A.1/A.2) into individual cassette cells using a funnel.

##### Estimating the carrying capacity of the insect cassette

To estimate the capacity of the insect cassette (versions A.1 and A.2), male *Ae. aegypti* were immobilized at 4 °C. Chilled males were then used to load five individual cells of the cassette. The contents of each cell were removed using a mouth aspirator and a fine brush and counted. The number of mosquitoes per cell was recorded, and the average number per cell for each insect cassette version was estimated.

##### Insect cassettes release uniformity and the number of males remaining in the cassette

The insect cassette's mosquito release uniformity (flow rate) was assessed at 4 °C by loading the cassette (version A.2) with 12,000 to 16,000 chilled male *Ae. aegypti* mosquitoes. The cassette was then embedded into the release system. The whole system was placed on a Sartorius® weighing balance (Satorius AG, Göttingen, Germany). The release mechanism was then activated by turning on the power switch of the insect release system. A videotape of the weighing balance display was made, showing the changes in mosquito weight over time (s) (Additional file 1: Video S1). The flow rate assessment was repeated once.

To estimate the number of mosquitoes that remained in the insect cassette version A.2 after release, chilled mosquitoes were loaded into 26 out of 38 cells at the centre of the cassette. Three cells in two rows (six cells) at each end of the cassette were left empty. After the release, the mosquitoes were brushed off and counted to estimate the number of remaining mosquitoes per cell.

##### Flight propensity of released male* Ae. aegypti* using the insect cassette

To estimate the flight propensity of released male *Ae. aegypti,* mosquitoes, the cassette was first filled with about 16,000 chilled mosquitoes. The insect release system was then placed on top of a containment box constructed of polymethyl methacrylate (PMMA) in the cold room (4 °C). A large Petri dish (diameter 18 cm) was used to collect chilled mosquitoes as they were released. Samples of mosquitoes were collected from the released mosquitoes to assess their flight propensity using the FAO/IAEA flight device [21, 26] in comparison to a control group (non-released). The release procedure was repeated twice with five replicates of approximately 100 mosquitoes each.

##### Survival rate of released male *Ae. aegypti* using the insect cassette

To evaluate the survival rate of male *Ae. aegypti* mosquitoes after release, three groups of 30 males each were collected immediately after their release in the cold room. Survival was assessed by providing the mosquitoes a 10% (w/v) sugar water solution for 27 days in 15 × 15 × 15-cm insect cages (BugDorm, MegaView Science Co., Ltd.) under adult rearing conditions. Dead mosquitoes were removed daily from the cages and counted, and the mortalities were compared to the control group (non-released laboratory male mosquitoes). The experiment was conducted twice with different batches of mosquitoes.

##### Impact of release on male *Ae. aegypti* damage

Physical damage was defined as missing legs (out of 6 legs) and missing/damaged wings. To estimate male *Ae. aegypti* physical damage, we collected three groups of 50 male mosquitoes each immediately after release in the cold room and compared these to three groups of control samples of 50 non-released males each. Male mosquitoes were kept at 4 °C for 2 days (less time may be needed for complete immobilization) prior to the assessment of physical damage.

#### Insect cassette suitability and release process impact assessment for *G. p. gambiensis*

##### Strain rearing and handling

Male *G. p. gambiensis,*, a major vector of human and animal trypanosomosis in West Africa [27], reared at the IPCL of the Joint FAO/IAEA Centre of Nuclear Techniques in Food and Agriculture (Seibersdorf, Austria), were used. This colony was initially established in 1972 with tsetse collected in Burkina Faso and reared in Maisons-Alfort, France [28]. The strain was subsequently transferred from France to the Centre International de Recherche-Développement sur l’Élevage en zone Subhumide (CIRDES) in Burkina Faso in 1975 and later established at the IPCL in 2009 [28]. The IPCL colony is maintained under standardized environmental conditions for tsetse colonies, at constant temperatures of between 24–25 °C and a RH of 75% ± 5%, with indirect illumination provided at a 12:12-h (light:dark) photoperiod [29]. The IPCL colony and tsetse used in the experiment were fed bovine blood 3 times a week using a defibrinated blood artificial membrane feeding system [29].

##### Estimating the carrying capacity of the insect cassette

To determine the total capacity of versions A.1 and B of the insect cassette, hand-sorted teneral male tsetse were chilled at 3.5 ± 1.0 °C until they became inactive and inserted into six random cells of the insect cassette until the cells were full. Care was taken not to compact the tsetse, and all tsetse that could fit when the cassette lid was closed were counted. After the tsetse were counted, they were divided into 10 groups of 100 each and weighed using a Sartorius® balance (Sartorius AG) to estimate the total weight of each version of the release system at full capacity.

##### Evaluation of the release proportions and the flight propensity of the released males

For the flight propensity assessments and insect cassette loading, 7-day-old males were first starved for 48 h and then chilled at 3.5 ± 1.0 °C until immobile using a chiller designed for standard tsetse colony maintenance [30]. The tsetse were then divided into four experimental groups for version A.1, which included three release groups and one control group, and into five experimental groups for version B, consisting of four release groups and one control group. For the three release groups of version A.1, three cells of the insect cassette, labelled A.1.1, A.1.2 and A.1.3, respectively (with A.1.1 positioned close to the front, at the end of the holding frame; A.1.2 positioned in, the middle; and A.1.3 positioned close to the reel; see Fig. 1) were selected to simulate the release of tsetse at three different time points. Each of the three cells was filled with 225 males, and the same number of tsetse were used for all the replications. For the control group of version A.1, 50 tsetse males were used for each replication. For the release groups of version B, four cells of the insect cassette (rows eight [B2] and eighteen [B4] in the left column and rows three [B1] and thirteen [B3] in the right column) were selected to simulate the release of tsetse at four different time points, and the same four cells were used throughout replicates. A total of 200 males was used per cell, for each replicate. For the control group of version B, 54 tsetse were used for each replication. Six replications were conducted for both versions A.1 and B.

The control groups for each insect cassette version were placed in a black polyvinyl chloride (PVC) flight cylinder, which was then positioned in the center of a 30 × 30 × 30-cm insect cage (BugDorm, MegaView Science Co., Ltd.) at 25 °C and 75 %RH immediately after they were removed from the chiller. The flight cylinders were 10 cm high with a diameter of 8 cm. The insides of the cylinders were coated with talcum powder to prevent tsetse from crawling out [31]. The control group was given 60 min for the tsetse to escape the flight cylinder. Afterward, the flight cylinder was covered and removed from the insect cage. All tsetse in the cage were categorized as flyers, while those remaining in the flight cylinder were classified as non-flyers.

For the release groups in the insect cassette, the prepared cassette was taken out of the chiller and allowed to acclimate for 20 min at 25 °C and 75% RH so the tsetse could recuperate before the machine was activated, initiating the release of the first group. The release system was positioned on a stand at a height of 15 cm in the center of a 60 × 60 × 60-cm insect cage (BugDorm, MegaView Science Co., Ltd.). As previously described, a flight cylinder was positioned directly beneath the insect cassette cells being released to capture all tsetse that fell from the exposed cell. A 60-min timer was started the moment the cell was exposed, allowing time for tsetse that fell inside the flight cylinder to escape. The insect cassette was turned off after the cell of the group being released was fully exposed. The cell was then blocked with tape to prevent non-released tsetse from crawling between cells or released tsetse from re-entering the open cells of the insect cassette. The insect cassette was then moved to an empty insect cage with the same setup for the release of the second group, simulating a new release time point. The same procedures were repeated for all release groups of both versions A.1 and B. After each 60-min timer ended, the flight tube was covered and removed from the corresponding insect cage with the finished timer.

Tsetse found in the insect cage and inside the flight tubes were regarded as released tsetse or flyers, while those still in the insect cassette were categorized as non-released tsetse. Individuals still inside the flight cylinder were classified as non-flyers. All non-flyers were observed for physical damage to their wings. The time recorded for both the control and released tsetse included their duration inside the chiller and the insect cassette.

### Statistical analysis

Data were statistically analysed using R studio version 4.4.2 (R Foundation for Statistical Computing, Vienna, Austria). The flight propensity between released and non-released *Ae. aegypti* mosquitoes was analysed using a generalized binomial linear mixed-effects model fit by maximum likelihood (Laplace approximation) with a logit link, with the proportion of flyers as the dependent variable and replicates as a random effect. The survival of mosquitoes following the treatment (2 levels: released and control) was analysed using mixed-effects Cox model (‘coxme‘ function in ‘survival’ package) fit by maximum likelihood with mosquito time to death as response variable, treatment (2 levels: released and control) as fixed effects and replicate as a random effect.

A similar binomial model as indicated above was used to analyse the percentage of tsetse released and the tsetse flight propensity, which were the model’s response variables. The explanatory variables were the insect cassette version, the experimental groups, chilling time, time the tsetse were in the cells of the insect cassette and the interaction between these variables. Replicates were used as random effects. The MuMIn package [32] was used to determine the Akaike information criterion corrected (AICc), and the model with the best value [33] was selected to explain the variation in the data. The ‘emmeans’ function from the emmeans package [34] was used for pairwise comparisons between the experimental groups.

## Results

### Insect cassette suitability and release process impact assessment for *Ae. aegypti*

#### Insect cassette carrying capacity

The average (± standard deviation [SD]) number of male *Ae. aegypti* that could fit into the individual cells of the insect cassette was 422.2 ± 28.3 for version A.2 and 2,258.2 ± 128.7 for version A.1. At a compaction density of 100 males/cm^3^, the average capacity of versions A.2 and A.1 is approximately 16,043.6 ± 1,076.9 and 45,164 ± 2,574.4, respectively. Based on the estimated average weight of one starved adult male *Ae. aegypti* (0.9 mg), the total weight of the fully loaded insect cassette was determined to be approximately 239.43 g for version A.2 and 265.64 g for version A.1.

#### Insect cassettes release uniformity and the number of male mosquitoes remaining in the cassette

The uniformity of mosquito release was assessed by weighing the total mosquitoes released over time. The equation used estimates the time required to release a known number of mosquitoes per unit area, relative to the drone speed (Additional file 1: Figure S1). For example, for an estimated male weight of 0.9 mg/male, the equation yields 11,928 male insects released after 7 min of flight.

Under laboratory conditions, an average of 96.47% ± 0.002% of the mosquitoes in the insect cassette were released after the simulated release using version A.2.

#### Estimating the flight propensity of released male *Ae. aegypti* mosquitoes using the insect cassette

**Fig. 3:**
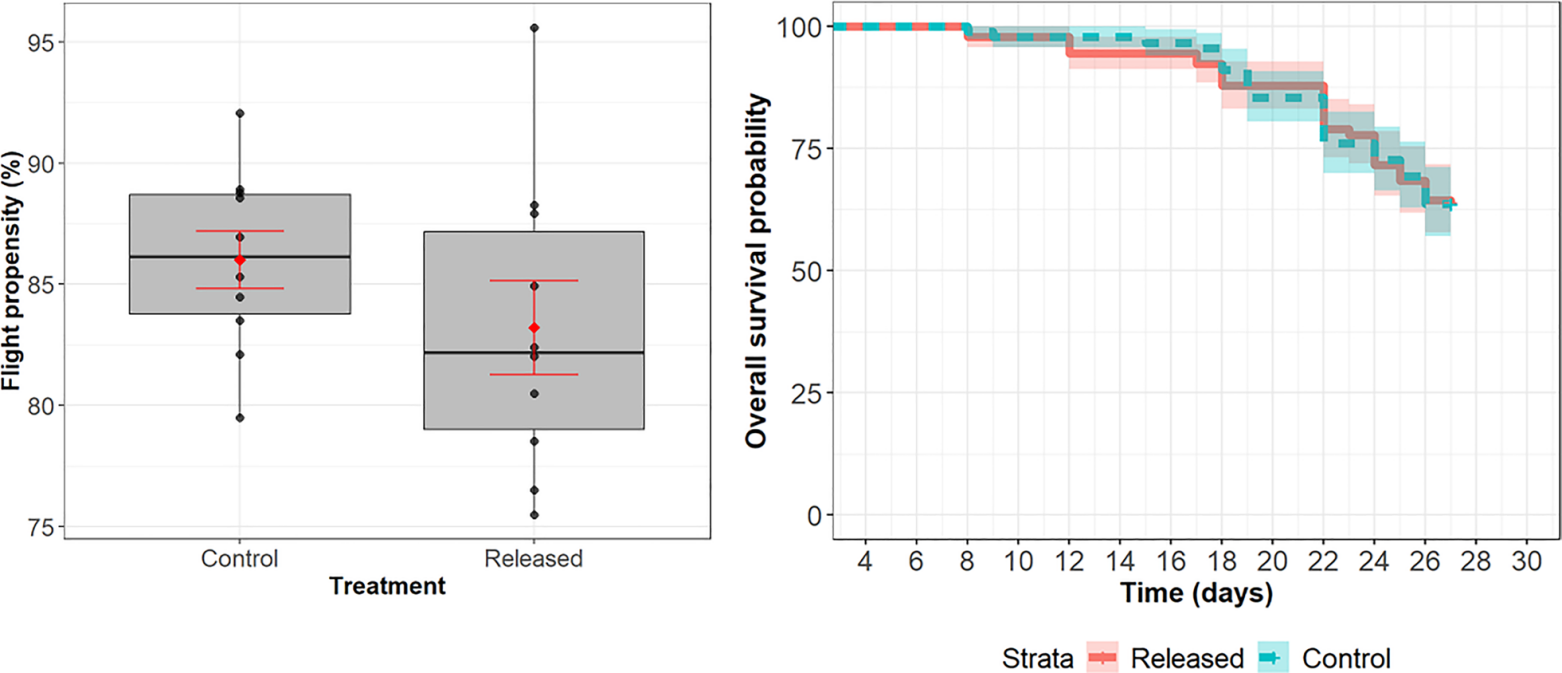
*Aedes aegypti* mosquito flight propensity according to treatments (control vs released males) (left) and overall survival probability according to the *Aedes aegypti* when released (solid line) or not (control) (dashed line) (right). The boxplot shows the median (thick horizontal line) as well as the upper and lower quartiles (upper and lower box boundaries); the means and standard errors are shown in red

The mean (± SD) flight propensity for the experimental mosquitoes did not differ significantly from that of the control group (83.0% ± 6.6% vs 86.1% ± 4.0%, respectively; (*χ*^2^ = 3.1661,* df* = 1, *P* = 0.07518) (Fig. 3 left), indicating that releasing *Ae. aegypti* mosquitoes using the insect cassette did not have a negative impact on flight propensity.

#### Estimating the survival rate of the released male *Ae. aegypti* mosquitoes using the insect cassette

Release from the insect cassette did not impact male *Ae. aegypti* survival as compared to non-released males in laboratory conditions (coefficient = 0.01572, * Z* = 0.09, *P* = 0.929) (Fig. 3, right). When mosquito survival was assessed, regardless of the treatment (released vs control), > 70% survived after a 26-day monitoring period in laboratory conditions (Additional file 1: Table S1).

#### Effect of release on damage to male *Ae. aegypti* mosquitoes

No substantial physical damage was observed in male mosquitoes after release. Of 150 mosquitoes released, only two had one or two missing legs (0.67–1.33%), and none had missing or damaged wings (0%).

### Insect cassette suitability and release process impact assessment for *G. p. gambiensis*

#### Insect cassette carrying capacity

An average (± SD) of 336.3 ± 13.4 male *G. p. gambiensis* per cell could fit in version A.1 of the insect cassette , giving an overall carrying capacity of 6726 ± 268 tsetse for all 20 cells; in version B, the average carrying capacity was 284.5 ± 17.0 tsetse per cell, giving an overall carrying capacity of 11,380 ± 680 tsetse for the 40 cells.

The average (± SD) weight of 1000 male *G. p. gambiensis* was 22.7 ± 0.3 mg. Taking this average weight into consideration, the estimated total weight of a fully loaded insect cassette was 378.1 ± 6.1 g in version A.1 and 571.0 ± 15.8 g in version B.

#### Evaluation of the release proportions and the flight propensity of released *G. p. gambiensis* males

For the release assays using the version A.1 insect cassette, 4059 tsetse were chilled for between 14 and 34 min before insertion into the three cells of the Insect cassette. For version A.1 of the insect cassette, the mean (± SD) time spent inside cells A.1.1, A.1.2 and A.1.3 was 21.8 ± 1.8, 27.5 ± 1.6 and 32.5 ± 1.6 min, respectively. The between-cell difference in the percentage of tsetse released was significant (*χ*^2^ = 9.7130,* df* = 2, *P* = 0.0078). The pairwise comparison between the cells indicated a significant difference for cell A.1.1 (67.8% ± 10.5%) compared to cell A.1.2 (61.7% ± 20.2%) and cell A.1.3 (64.7% ± 14.8%) (Fig. 3) (Additional file 1: Table S2).

**Fig. 4:**
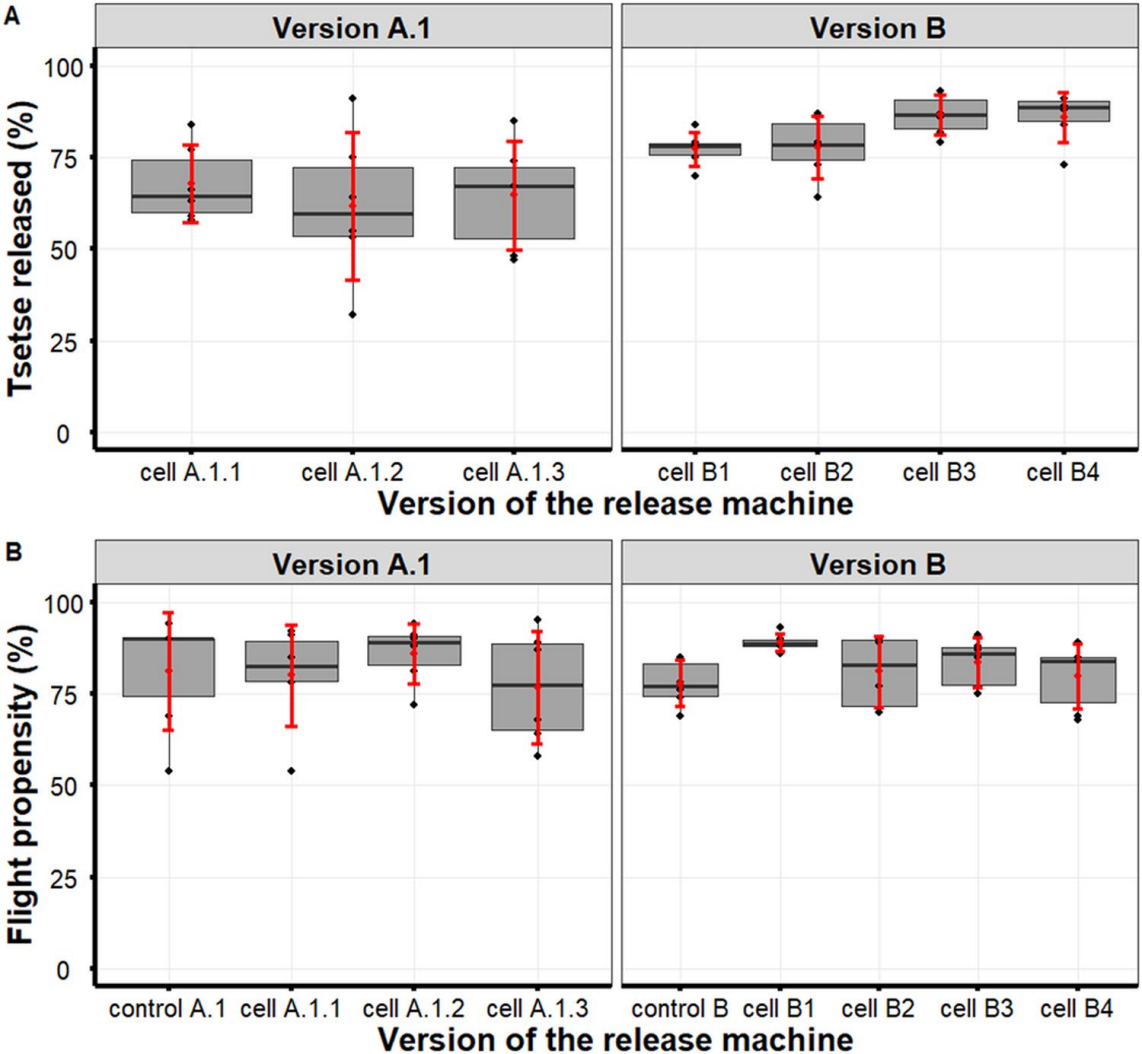
Percentage of *Glossina palpalis gambiensis* that were released from each cell of version A.1 and B of the insect cassette (top row) and flight propensity as determined by the *G. p. gambiensis* flyers that escaped the flight cylinder after being released from the cells of the two insect cassette versions A.1 and B (bottom row). The boxplot shows the median (thick horizontal line) as well as the upper and lower quartiles (upper and lower box boundaries); the means and standard errors are shown in red

Of the tsetse that were released, the mean (± SD) flight propensity of those that could escape the insect cassette and flight cylinder was 80.0 ± 13.9% for cell A.1.1, 86.0 ± 8.1% for cell A.1.2 and 76.8 ± 15.4% for cell A.1.3 (Fig. 4). For the control flies, the mean flight propensity was 81.2 ± 16.0%. No significant difference in flight propensity was observed between the groups for version A.1 of the insect cassette overall (*P* > 0.05) (Additional file 1: Table S3). Pairwise comparisons indicated no significant difference between the tsetse that were released from cell A.1.2 and the flies released from cells A.1.1 and A.1.3 (*χ*^2^ = 20.256,* df* = 3, *P* = 0.0002) (Additional file 1: Table S3). No physical damage was observed in the non-flyers (tsetse that were not able to escape the flight cylinder).

The four cells of insect cassette version B were loaded with 4800 tsetse that had been chilled for 22 to 31 min. The mean (± SD) time the tsetse spent inside cells B.1, B.2 and B.3 of the version B insect cassette was 30.5 ± 12.0, 39.3 ± 12.1, 47.8 ± 12.2 and 57.2 ± 12.6 min, respectively. Similar to version A.1, a significant difference in the percentage of tsetse released between the cells (*χ*^2^ = 58.798,* df* = 3, *P* < 0.0001) was also observed for version B. There was no significant difference in the percentage of tsetse released from cell B.1 (77.3% ± 4.6%) compared to B.2 (77.8% ± 8.6%), and from cell B.3 (86.5% ± 5.5%) compared to B.4 (86.0% ± 6.9%), as indicated by the pairwise comparison (Fig. 3; Additional file 1: Table S2). However, there was a significant difference in the release percentage from cell B.1 compared to cells B.3 and B.4 as well as between cell B.2 compared to cells B.3 and B.4. The mean flight propensity of the tsetse that were released from cells B1, B2, B3 and B4 were 89.0% ± 2.4%, 81.0% ± 9.8%, 83.5% ± 6.9% and 79.8% ± 9.0%, respectively. For the control flies, the mean flight propensity (77.8% ± 6.3%) was lower than that of the released tsetse. There was a significant difference between the released and control groups (*χ*^2^ = 38.419,* df* = 4, *P* = 9.185e-08). The pairwise comparison indicated a significant difference in flight propensity for tsetse released from cell B1 (89.0% ± 2.37%) compared to those released from cell B2 (81.0 ± 9.84%), cell B3 (83.5 ± 6.86%) and the control (77.83 ± 6.31%) (Additional file 1: Table S3). There was no significant difference in flight propensity for the tsetse released from cell B2 (81.0% ± 9.84%), cell B3 (83.5% ± 6.86%) and cell B4 (79.83% ± 8.98%) compared to the control and each other. No physical damage was observed in either the flyers or the non-flyers.

Finally, when comparing the proportion of tsetse released (*χ*^2^ = 328.01,* df* = 1, *P* < 0.0001) from versions A.1 and B of the insect cassette and their flight propensity (*χ*^2^ = 52.66,* df* = 6, *P* < 0.0001), a significant improvement was observed for each parameter.

## Discussion

The SIT suppresses insect populations by releasing sterilized males into a target area, thereby preventing/limiting successful reproduction. This technique has been successfully used against agricultural pests and has been adapted to vectors such as mosquitoes and tsetse. Notably, SIT eradicated *G. p. gambiensis* in Senegal and reduced *Ae. aegypti* densities in field trials, with success relying on uniform, large-scale releases. The use of insect cassettes and drones to release sterile male mosquitoes and tsetse represents a significant advancement in the implementation of SIT and other vector control strategies. Our study assessed key performance indicators, such as carrying capacity of the cassette and release uniformity, flight propensity and survival of the target species, to evaluate the suitability of a novel 3D-printed insect cassette release system.

We found that both cassette versions tested exhibited sufficient carrying capacity for *Ae. aegypti* and *G. p. gambiensis*, with version B showing superior capacity for tsetse. While release percentage was tested for only A.2 (for *Ae. aegypti*), we observed almost complete release. Version B achieved high mean release percentage overall as compared to A.1 when tested with tsetse (Additional file 1: Table S4). The lightweight and portable nature of this insect cassette version offers notable logistical advantages. Specifically, the capacities of versions A.1 and A.2 were approximately 45,164 and 16,043 male *Ae. aegypti* mosquitoes, respectively, whereas versions A.1 and B could carry up to 6726 and 11,380 *G. p. gambiensis*, respectively. This aligns with findings reported by Lin et al. [19], who described a release mechanism comprising four mosquito storage canisters, each capable of carrying approximately 40,000 adult mosquitoes. Similarly, the Mubarqui Smart Release Machine 2 (MSRM2) and the Bruno Spreader Innovation (BSI) systems, both operated using gyrocopters, offer carrying capacities of around 15,000 *G. p. gambiensis*, although these systems are significantly heavier at 64 and 20 kg, respectively [14, 15]. In another study, a different mosquito release system canister filled with 50,000 marked mosquitoes was used in a release trial from an uncrewed aerial vehicle in Brazil [17]. All of these systems include a chilling option [25] to maintain insect quality during releases [13, 15]. While the capacity of the insect cassette is slightly lower than that of the MSRM2, the BSI systems or the systems described by Bouyer et al. [17] and more recently by Lin et al. [19], respectively, and it does not include a chilling option, its reduced weight (< 2 kg) and modular design make it highly suitable for drone-based applications where payload limitations are critical. However, the lack of integrated chilling mechanism may limit the duration of releases or the resilience of insects under higher ambient temperatures. These results indicate that the insect cassette has a sufficiently practical size for operational use, particularly in programmes where drones are the primary release platform. Additionally, the design of the drone-compatible release mechanism described by Bouyer et al. [17] showed that capacity optimization plays a pivotal role in achieving operational scalability and maintaining insect quality during aerial releases.

Uniformity in releasing sterile males is also critical for maintaining the sterile-to-wild male ratio across target areas. Our results indicated consistent and uniform distribution patterns for *Ae. aegypti*, a feature demonstrated in previous studies. For example, Bouyer et al. [17] also emphasized the importance of precise release uniformity in enhancing SIT intervention success by reducing the risk of localized over- or under-dispersion. Furthermore, the automated chilled release system tested by Mirieri et al. [15] achieved homogeneous dispersal of sterile male tsetse, ensuring their competitiveness and effectiveness. Although in the present study the release was simulated in the laboratory, 96% of the insects were successfully released. This demonstrates the performance of the insect cassette even in the absence of wind and vibrations. However, differences in insect sizes may play a role in the design and efficacy of release cassettes. This precision matches observations in other aerial release systems where homogeneous distribution improved the competitiveness and efficacy of sterile males.

Once released, insects should demonstrate their ability to fly, an essential feature for the success of the SIT. Our findings indicated that the flight performance of both species post-release was minimally affected, reinforcing the conclusions of Bouyer et al. [17] that drone-based release systems can maintain insect quality during dispersal. The integration of drones with release systems significantly enhances the efficiency of SIT and other release-based programmes [19]. Our study provides actionable insights into operational planning by estimating the time required to release mosquitoes per unit area relative to drone speed. For example, with an estimated weight of 0.9 mg per male, the equation results in the release of > 10,000 male insects after 7 min of flight. Using a drone with a cruise speed of 5 m/s, a total distance of 2100 m could be covered within 7 min, considering the drone's line of sight. This efficiency aligns with findings reported by Mubarqui et al. [14], who highlighted that drone-compatible systems reduce operational costs and improve accessibility to remote areas. Furthermore, our study results emphasize the importance of species-specific dispersal behaviours in optimizing aerial release strategies, ensuring that released males effectively reach target populations.

Devices such as the MSRM2 and the BSI have demonstrated the potential for automated systems to revolutionize aerial insect releases. The MSRM, which was tested for fruit flies and tsetse, achieved high dispersal homogeneity and adaptability to various release densities. Similarly, the BSI system showed that automated chilled releases could ensure consistency in insect quality while addressing logistical challenges such as transportation and environmental control. At a drone speed of approximately 19 m/s, the estimated flight time for a typical release operation is around 40 min. Our findings demonstrate that *G. p. gambiensis* held inside the cassette for up to 57 min at 25 °C exhibited no significant reduction in flight propensity compared to control groups not subjected to release. However, in Senegal, the outside temperature can reach 30–40 °C, and further tests are thus needed to assess how such temperatures would affect the quality of the released insects. These advancements underscore the relevance of continuous innovation in release technologies to address species-specific requirements and operational constraints.

The findings of this study contribute to a growing body of evidence supporting the integration of drone-based systems in SIT programmes. The technology offers numerous advantages, including reduced costs (as compared to acquisition and maintenance costs of aircraft, fuel costs, gyrocopter pilot hiring), minimized environmental impact and enhanced precision in dispersal [16, 20, 35]. Bouyer et al. [17] and Mubarqui et al. [14] both noted that drone systems could effectively scale-up SIT programmes while maintaining ecological safety. Additionally, the ability to access restricted or remote areas with drones addresses a significant limitation of traditional fixed-wing aircraft [35].

While the laboratory conditions provided controlled insights into the insect cassette performance, several limitations to our study should be acknowledged. First, as the evaluation was conducted only in laboratory settings, field validation is essential to assess how environmental factors such as temperature fluctuations, humidity, wind exposure and release-related vibrations may affect insect survival and release uniformity. Second, in the present study we assessed release homogeneity and insect quality in mosquitoes under chilled conditions, whereas the cassette lacks a chilling mechanism. This difference highlights the need to optimize the device design for temperature control during drone operations. Third, we used non-irradiated insects, whereas SIT programmes rely on irradiated males that may be more sensitive to stressors such as friction or vibrations [25, 31]. Consequently, more research is needed to assess cassette performance with irradiated insects under simulated operational conditions. Finally, future studies should focus on optimizing cassette design, integrating a chilling mechanism to enhance durability and capacity while ensuring insect quality, as suggested by Mubarqui et al. [14]. Also, expanding the use of such systems to other vector species or ecological zones could validate their versatility and the impact of release-based programmes globally.

## Conclusions

Overall, our findings suggest that the insect cassette is a promising prototype for drone-based aerial release programmes targeting mosquitoes and tsetse. While the results demonstrate its potential under controlled conditions, further research is needed to refine the design and functionality of the system. Evaluating its performance in field settings, such as field competitiveness and mark-release-recapture studies, to validate its effectiveness in vector control will be essential. Moreover, assessing the cost-effectiveness and scalability of the system compared to traditional release methods will be vital for guiding policy decisions and resource allocation in vector control programmes.

## Supplementary Information

Additional file 1 (XLSX 18 kb)

Additional file 2 (MP4 92057 kb)

Additional file 3 (MP4 2954 kb)

## Data Availability

Data supporting the main conclusions of this study are included in the manuscript.
